# Alterations in Sensitivity to Estrogen, Dihydrotestosterone, and Xenogens in B-Lymphocytes from Children with Autism Spectrum Disorder and Their Unaffected Twins/Siblings

**DOI:** 10.1155/2013/159810

**Published:** 2013-11-06

**Authors:** Martyn A. Sharpe, Taylor L. Gist, David S. Baskin

**Affiliations:** Department of Neurosurgery, The Methodist Neurological Institute, 6560 Fannin Street, Scurlock Tower 944, Houston, TX 77030, USA

## Abstract

It has been postulated that androgen overexposure in a susceptible person leads to excessive brain masculinization and the autism spectrum disorder (ASD) phenotype. In this study, the responses to estradiol (E2), dihydrotestosterone (DHT), and dichlorodiphenyldichloroethylene (DDE) on B-lymphocytes from ASD subjects and controls are compared. B cells were obtained from 11 ASD subjects, their unaffected fraternal twins, and nontwin siblings. Controls were obtained from a different cell bank. Lactate dehydrogenase (LDH) and sodium 2,3-bis(2-methoxy-4-nitro-5-sulfophenyl)-2H-tetrazolium-5-carboxanilide (XTT) reduction levels were measured after incubation with different concentrations of E2, DHT, and DDE. XTT/LDH ratio, representative of mitochondria number per cell, was calculated. E2, DHT, and DDE all cause “U”-shaped growth curves, as measured by LDH levels. ASD B cells show less growth depression compared to siblings and controls (*P* < 0.01). They also have reduced XTT/LDH ratios (*P* < 0.01) when compared to external controls, whereas siblings had values of XTT/LDH between ASD and external controls. B-lymphocytes from people with ASD exhibit a differential response to E2, DHT, and hormone disruptors in regard to cell growth and mitochondrial upregulation when compared to non-ASD siblings and external controls. Specifically, ASD B-lymphocytes show significantly less growth depression and less mitochondrial upregulation when exposed to these effectors. A mitochondrial deficit in ASD individuals is implied.

## 1. Introduction

Autism spectrum disorder (ASD) is a developmental disorder characterized by abnormal communication, social impairment, and stereotyped restricted interests and behavior. There has been a 10-fold increase in the incidence of ASD over the last two decades, with ASD now affecting 1 in 88 US children [1, 2]. ASD has a 5 : 1 male to female gender bias and is usually diagnosed before 4 years of age. Other male biased conditions [3] including dyslexia [4], specific language impairment, ADHD, and oppositional defiant disorder (ODD) have also increased during the last few decades [3, 4]. The cost of supporting people with ASD in Great Britain, including the cost of lost productivity, is *£*2.7b/year for children and *£*25b/year for adults [5]. Medical expenditures on individuals with ASD in the US are 4.1–6.2× greater than for those without, with average annual healthcare expenditures of approximately $6000/child reported and precipitously rising [6, 7]. Phenotypic analyses of male biased cognitive disorders suggest that genetic influences increase susceptibility to multiple cognitive deficits [8–10]. The disease triggers and temporal window(s) in susceptible individuals are unknown, although many have postulated exposure to environmental chemical and/or toxins as candidates.

### 1.1. Philosophy for the Experimental Design

Fetal androgen exposure can alter responses to estrogens/androgens in later life with profound results. Individuals with ASD have a cognitive empathy deficit, and women have higher scores in tests of cognitive empathy than do men. Women exposed to supraphysiologic androgen concentrations show a decline in cognitive empathy [11]. However, the degree to which cognitive empathy is “masculinized” is greatest in women who have a low 2D : 4D ratio, a marker of *in utero *androgen exposure. Manning et al. proposed that the ratio of the second-to-fourth finger length, the 2D : 4D ratio, was a marker for prenatal androgen action [12]. Thus, if the 2D : 4D ratio is a true proxy for fetal androgen exposure, then the “masculinized” brain remains under the control of androgen throughout life. Estrogen alters mitochondrial state 3 respiration [13], and many aspects of mitochondria function and biogenesis are under estrogenic control [14, 15], especially (rat) brain mitochondria [16]. Androgens are known to change the levels of mitochondria in many tissues [17]. Breast lactate dehydrogenase-*α* (LDH*α*) is upregulated by 17*β*-estradiol (E2) [18], but in Sertoli cells both E2 and dihydrotestosterone (DHT) downregulate LDH*α* expression and lactate generation while increasing glucose consumption [19], suggesting a switch from anaerobic to aerobic respiration. With respect to ASD, we should be able to observe a differential response to androgens/estrogens and perhaps to hormone mimetics in cells that have been presensitized *in utero*.

A number of groups have been attempting to understand the genetics of ASD [21] and the impact of rare copy number variants [22]. In recent studies, the expression profiles of B-lymphocytes from ASD individuals and age-matched controls were analyzed by cDNA microarrays [23, 24]. Investigators have found that the major pathways differentially affected involved steroid hormone biosynthesis. Further, steroid hormone and neurotransmitter pathways formed the bulk of the differentially expressed genes. This is interesting, considering that in some studies the ASD population has higher testosterone levels than their siblings [24]. Blood samples from subjects with ASD and their siblings are thus an important resource for examining genetic/environmental interactions on cell development. B cells express both estrogen receptor-*α* (ER*α*; both the 46 and 66 isoforms) and estrogen receptor-*β* (ER*β*) receptors [25], and androgens markedly alter IgG/IgM expression in mature B-lymphocytes [26]. B cells also have the ability to transform estrogens/androgens to neurosteroids [27].

### 1.2. DDT/DDE

The pesticide dichlorodiphenyltrichloroethane (DDT; banned in US since 1972) and its primary metabolite dichlorodiphenyldichloroethylene (DDE) remain potential hazards due to their worldwide use, long half-life, and resultant accumulation in the food chain. DDE, which binds to both ER*α* and ER*β*, has both androgenic and antiandrogenic activities. In addition, the levels of ER*α* and ER*β* in human breast cancer correlate with serum DDE levels [28], indicating that cancer cells can be sensitized to estrogenic signals by DDE. DDE also mimics the “U”-shaped E2 stimulation/repression titration curve of *β*-hexosaminidase release in these mast cells over a 0.1–100 nM range [29].

### 1.3. Environmental Androgen/Estrogen Agonists/Antagonists and ASD

The eightfold increase in children born in California with ASD since 1990 cannot be attributed simply to changes in diagnostic criteria or record-keeping, suggesting a combination of genetic predisposition and environment [30]. Anthropogenic endocrine disruptors are man-made chemicals that interfere with the body's endocrine system and produce adverse effects in both humans and wildlife. A wide range of substances are thought to cause endocrine disruption, including pharmaceuticals, dioxin/dioxin-like compounds, polychlorinated biphenyls, DDT and its primary metabolite DDE, and plasticizers such as bisphenol A [31]. An investigation in Sweden has also shown that ASD appears to be linked to phthalate exposure early in development, resulting from the usage of PVC flooring [32].

### 1.4. Hypothesis

In this study, the responses to E2, DHT, and DDE, and other hormone disruptors on immortalized B-lymphocytes from ASD subjects and controls are compared. Lymphocytes are easy to obtain and culture but are clearly not present in brain under normal circumstances. Knowing very well that ASD is a disorder with profound neurological sequelae and that neuronal and/or glial cells are almost certainly involved or affected, normal human astrocytes and cortical neurons were also examined in the presence of the aforementioned agents. We postulated that B cells from ASD individuals would have a different proliferative response to steroid hormones and other hormone disruptors, and normal human astrocytes and cortical neurons would have responses similar to control B-lymphocytes. As we cannot easily study astrocytes and cortical neurons from individuals with autism, we believe it is important to compare the results of responses of normal human astrocytes and neurons to normal control B-lymphocytes to see if the response is similar.

## 2. Methods

### 2.1. B-Lymphocyte Populations

B-lymphocytes were obtained from 11 ASD subjects (AUT) from the Autism Genetic Resource Exchange (AGRE) tissue bank who had an unaffected fraternal twin and another unaffected sibling. This included 10 male and 1 female AUT, along with 11 brothers (Bro) and 11 sisters (Sis). The fraternal twins of the 10 male AUT consisted of 4 brothers (Twin Bro) and 6 sisters (Twin Sis). B-lymphocytes of 11 individuals from a different cell bank (Coriell Institute depository, controls for a longitudinal study of obesity) were used as an external control (Con). The Con were sex and age matched to the AUT subjects but had no known personal or family history of ASD. The growth of these cells was examined after 5 days of incubation with different concentrations of E2, DHT, and DDE using two assay systems, lactate dehydrogenase and mitochondrial sodium  2,3-bis(2-methoxy-4-nitro-5-sulfophenyl)-2H-tetrazolium-5-carboxanilide (XTT) reduction. We measured total LDH levels as a proxy of cell numbers [33] and XTT reduction as a proxy of mitochondrial reductase levels [34]. We validated that LDH levels correlated with cell numbers by selective counting using a Countess cell counter (Invitrogen, Carlsbad, CA, USA). Researchers were blinded to the identity of all cell families until the conclusion of all data acquisition.

### 2.2. Astrocyte and Neuron Populations

Normal human astrocytes (NHA) were obtained from Lonza (Walkersville, MD, USA) and human cortical neurons (HCN) from the ATCC (American Type Culture Collection, Manassas, VA, USA) and grown subject to their recommendations. NHA were grown in Astrocyte Cell Basal Medium supplemented with 3% FBS, glutamine, insulin, fhEGF, GA-1000, and ascorbic acid. HCN were grown using ATCC-formulated Dulbecco's Modified Eagle's Medium (Cat no. 30-2002) and supplemented with 10% FBS. NHA were grown to confluency in the appropriate media on Costar 96-well growth plates (Corning, NYC, NY, USA), and HCN were grown on 16-well Lab-Tek slide chambers (Nalge Nunc, Rochester, NY, USA). Cells were grown for 4 days in the presence of effectors at the following concentrations: 1.2 nM E2, 12 nM DHT, and 50 nM DDE. LDH and XTT reduction assays were then performed as detailed below.

### 2.3. LDH Assay

50 *μ*L of cells was assayed for the levels of lactate dehydrogenase activity in the presence of detergent [35, 36]. The final assay mixture comprised 110 mM lactate, 3.35 mM NAD^+^, 350 *μ*M resazurin, and 2.2 units/mL of diaphorase in 3 mM Tris/30 mM HEPES/10 mM NaCl buffer (pH 7.4) and 0.45% Triton X-100. The resorufin formed was measured in a plate reader using 530/25 nm ex and 590/35 nm em. The rates at which resorufin is formed are proportional to the levels of LDH.

### 2.4. XTT Assay

50 *μ*L of cells was withdrawn to be assayed for mitochondrial function/number using the XTT (2,3-bis(2-methoxy-4-nitro-5-sulfophenyl)-2H-tetrazolium-5-carboxanilide) mitochondrial and extramitochondrial dehydrogenases assay method [37, 38]. The cells were added to 50 *μ*L of 1 mg/mL XTT and diluted in growth media. The XTT converts to formazan by mitochondrial reductase which is only active in metabolically intact cells. After 60 minutes of incubation, 100 *μ*L of stop solution (40% SDS in 1 : 1 water : ethanol) was added, and the formazan product is measured at 570 minus 650 nm. A cell-free media control was treated in the same manner to afford the baseline level of XTT reduction.

### 2.5. Stripped and Unstripped Media

Preliminary experiments demonstrated the importance of using charcoal stripped fetal calf serum in the growth of B cells and human cortical neurons (HCN). Samples of stripped and unstripped serum were sent to two independent laboratories for hormonal (Texas Veterinary Medical Diagnostic Laboratory, College Station, TX, USA) and pesticide (Environmental Micro Analysis, Inc., Woodland, CA, USA) analysis. Pesticide levels in both unstripped and stripped fetal calf serum were below the detection threshold of the test procedures. Significant levels of steroidal hormones were found in unstripped media, and these were greatly lowered by charcoal stripping (Table 1). The differences in growth between stripped and unstripped media suggest that androgens and/or estrogens in the fetal calf serum may retard cell proliferation. Stripped serum was thus used in all the subsequent experiments reported herein.

### 2.6. Other Xenoestrogen/Androgen Hormone Disruptors

A large number of man-made compounds have been implicated as hormone disruptors, and we wished to examine if there was a differential sensitivity of AUTs to these compounds. A range of compounds previously suspected of altering estrogen/androgen signaling in mammalian cells were screened. Di-2-ethylhexyl phthalate (Phthalate DEHP), nonylphenol, bis-phenol A, and hydroxylated polychlorinated biphenyl (HO-PCB) were chosen based upon their wide usage and previous studies implicating these agents as hormone disruptors. Four randomly chosen male AUTs and their sex matched controls B-lymphocytes were exposed to these agents, and LDH and XTT assays were performed as described above.

## 3. Results

**Figure 1:**
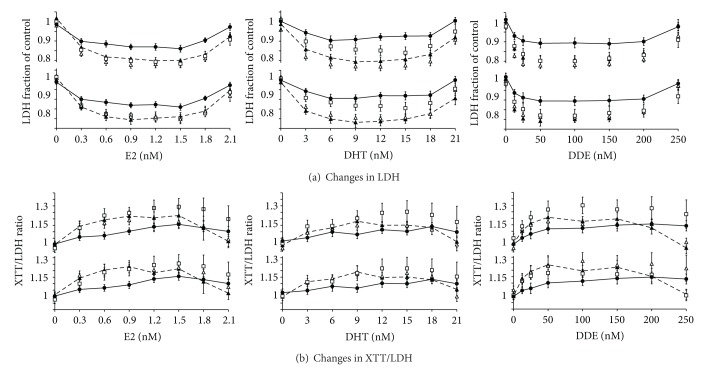
Changes in growth of B-lymphocytes and in their mitochondrial/cell ratio as a function of E2, DHT, and DDE concentrations. The figure shows the changes observed in B cells grown in the presence of E2, DHT, and DDE. The upper part of each panel shows the populations sorted by gender, with the 10 male AUT (*⚫*), 10 Con (□), 10 Bro (▲), and 10 Sis (∆). The lower part of each panel shows the populations sorted by birth relationship, with the average of 11 AUT (*⚫*), 11 Con (□), 11 Twin (▲), and 11 Sib (∆). The solid and dashed lines represent the AUT and Bro/Twin plots, respectively. (a) shows change in growth of B cells (LDH levels), and (b) shows change in the mitochondria/cell ratio (XTT/LDH), after five days in presence of effectors at listed concentrations as compared to controls. The solid line connects the AUT and the broken line the Bro and Twin, respectively. Error bars represent SEM. ASD: autism spectrum disorder; AUT: B cells from individual with ASD; Bro: B cells from brother of individual with ASD; Con: control B cells from individual with no personal or family history of ASD; DDE: dichlorodiphenyldichloroethylene; DHT: dihydrotestosterone; E2: estradiol; LDH: lactate dehydrogenase; SEM: standard error of the mean; Sib: B cells from phenotypically normal male or female siblings of individual with ASD; Sis: B cells from phenotypically normal sister of individual with ASD; Twin: B cells from phenotypically normal fraternal twin of individual with ASD; XTT: 2,3-bis(2-methoxy-4-nitro-5-sulfophenyl)-2H-tetrazolium-5-carboxanilide; XTT/LDH: ratio of XTT/LDH indicative of mitochondrial function per cell.

**Figure 2:**
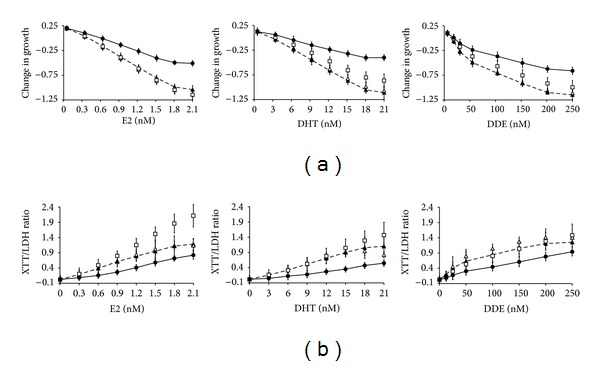
Changes in growth of B-lymphocytes and their mitochondrial/cell ratio as a function of E2, DHT, and DDE concentrations. Cumulative sum plots of Figure 1 data were created showing the effects of the 3 effectors on the 4 cell types, AUT (*⚫*), Bro (▲), Sis (∆), and Con (□). Solid line = AUT, broken line = Bro. Error bars are SEM. (a) shows changes in cellular growth as evidenced by LDH levels in the different cell populations when grown in the presence of the 3 effectors. (b) shows changes in the mitochondrial function per cell as evidenced by the XTT/LDH ratio. The starting concentration of zero nM represents the baseline LDH and XTT/LDH levels normalized to 0. The most significant difference between the AUT and Con cells in regard to growth suppression and mitochondrial function was seen in the presence of DHT. ASD: autism spectrum disorder; AUT: B cells from individual with ASD; Bro: B cells from brother of individual with ASD; Con: control B cells from individual with no personal or family history of ASD; DDE: dichlorodiphenyldichloroethylene; DHT: dihydrotestosterone; E2: estradiol; LDH: lactate dehydrogenase; SEM: standard error of the mean; Sis: B cells from phenotypically normal sister of individual with ASD; XTT: 2,3-bis(2-methoxy-4-nitro-5-sulfophenyl)-2H-tetrazolium-5-carboxanilide; XTT/LDH: ratio of XTT/LDH indicative of mitochondrial function per cell.

B cells derived from individuals with ASD (AUT) react differently to the hormones/hormone mimetics tested (i.e., E2, DHT, and DDE) by exhibiting less growth suppression and less mitochondrial proliferation when compared to all other cell populations tested (Figures 1 and 2). Two-way ANOVA analysis showed these differences to be statistically significant (*P* < 0.0001). We found that B cells derived from individuals with ASD have shallower U-shaped growth curves compared to the other B-cell populations when exposed to E2, DHT, and DDE. This indicates that AUT B cells show less growth depression as compared to non-AUT B cells in the presence of these agents. All growth titrations show the classical “U”-shaped (nonmonotonic dose response) curves associated with estrogens, androgens, and pesticide hormone mimetics. Only a small proportion (<1%) of the receptor pool need to be occupied by ligand to initiate a response, and so activation occurs at low hormone levels. At high hormone levels, signals may fall via receptor downregulation [39, 40]. The most significant effect was seen with DHT, where AUT cells showed the least growth suppression and mitochondrial upregulation (see the middle columns of Figures 1 and 2 as compared to the right and left columns).

### 3.1. Changes in Growth due to Effectors in the Four Cell Populations

In terms of growth suppression, AUT are significantly different from unaffected brothers and sisters and from age/sex matched cells taken from the general, unaffected, population. In all cases where AUT are compared with either internal or external controls, the cumulative sum plots actual observed slopes' confidence intervals falling outside the theoretical critical slopes' confidence intervals (Table 2). If there was no difference between the populations, we would expect to see overlap in the confidence intervals between the actual and theoretical slopes. Thus, there is a <1% chance that the AUT cell population is part of the general population of controls. In all cases where internal and external controls are compared, there is overlap between the actual and theoretical slopes' confidence levels. Thus, all internal and external controls appear to represent a single population.

### 3.2. Mitochondrial Effector Responses in the Four Cell Populations

In Table 3, we compare the changes in the XTT/LDH ratio in the form of the cumulative sum plots shown in Figure 2. AUT are again statistically different from the external controls, this time with respect to their ability to upregulate mitochondrial numbers (i.e., XTT/LDH ratio) in response to effectors. However, the response of the internal controls (i.e., Bro and Sis) straddles the divide of the AUT and Con population. Cells from the general population are able to increase their mitochondrial levels, while cells from autistics show a mitochondrial regulatory deficit, and the brothers and sisters of autistics represent a middle-ground between the two. In summary, Tables 2 and 3 show that B-lymphocytes from autistic subjects can be characterized as having poor growth suppression in response to authentic hormones and DDE and being less able to increase their mitochondrial numbers in response to E2, DHT, or DDE.

### 3.3. Effects on Astrocytes and Neurons

**Figure 3:**
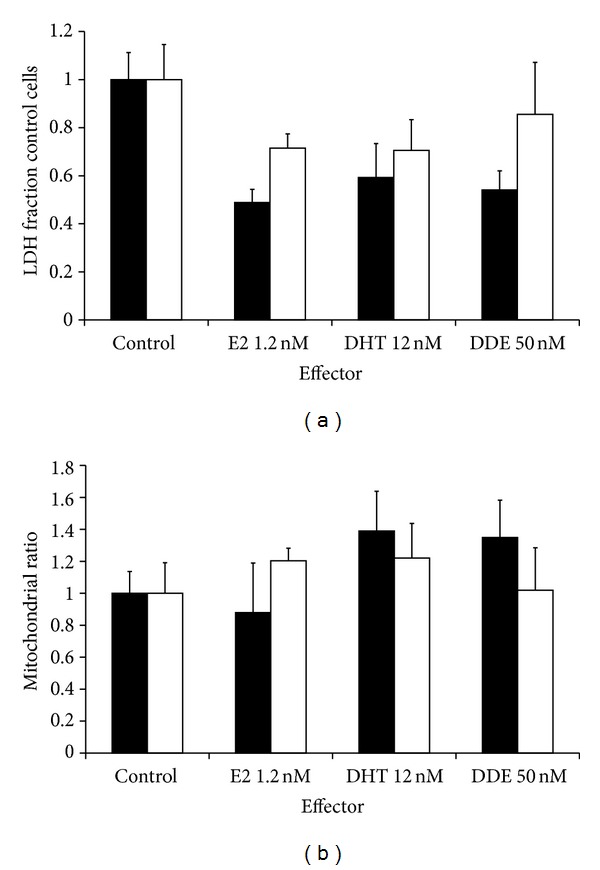
Effects of E2, DHT, and DDE on normal human astrocyte and human cortical neuron growth and XTT/LDH ratio. The figure shows the changes in normal human astrocyte (black bars) and human cortical neuron (white bars) growth (LDH level, left column) and the XTT/LDH ratio (right column) over four days in the presence of 1.2 nM E2, 12 nM DHT, and 50 nM DDE. Each bar represents 15 individual wells of cells and the error bars are the SD. E2, DHT, and DDE inhibit astrocytic and neuronal cell growth and cause changes in mitochondrial levels. DDE: dichlorodiphenyldichloroethylene; DHT: dihydrotestosterone; E2: estradiol; LDH: lactate dehydrogenase; SD: standard deviation; XTT: 2,3-bis(2-methoxy-4-nitro-5-sulfophenyl)-2H-tetrazolium-5-carboxanilide; XTT/LDH: ratio of XTT/LDH indicative of mitochondrial function per cell.

Based on the data we obtained using lymphocytes, we examined the effects of the three effectors on the growth and the mitochondrial numbers of normal human astrocytes (NHA) and human cortical neurons (HCN). We find effects on both growth and in the XTT/LDH ratio in both cell types at concentrations which cause differential effects in AUT/control B-lymphocytes (Figure 3). All three effectors cause a statistically significant (*P* < 0.05) drop in the growth rate of both human astrocytes and neurons. Mitochondria numbers were increased in human cortical neurons by both E2 and DHT, but not DDE (*P* < 0.05). Conversely, upregulation of human astrocytic mitochondria numbers occurred following incubation with DHT and DDE, but not E2, again at *P* < 0.05. Long known to be neuroprotective, E2 stimulates astrocytes to release growth factors, at the expense of proliferation [41]. The neurons show a 20% increase in their mitochondrial numbers when exposed to E2, which replicates the effects that E2 had on B cells. Androgens have been shown to induce MAPK signaling in neurons that subsequently drives neuroprotection, protecting them from mitochondrial disruption [42, 43] and amyloid peptide toxicity [44]. The two cell types have a differential response to DDE: in astrocytes growth drops and mitochondrial levels rise, but neurons show no statistically significant changes to exposure. It should be noted that these cells were not titrated with these effectors, and it is possible that astrocytes and neurons have different growth and mitochondrial response profiles to those observed in B-lymphocytes.

### 3.4. Other Hormone Disruptors

Table 4 shows the effects of the other hormone disruptors on AUT and Con B cells along with E2, DHT, and DDE. The major difference between these four hormone disruptors and the authentic hormones or DDE was the titration curve shape of both growth and the XTT/LDH ratio. The growth curves were more “L”-shaped than “U”-shaped, and instead of KD_2_ being 10–20 times larger than KD_1_, it was typically 10,000 to 100,000 times larger. In the titrations, there was always a slight upswing after the maximal change in amplitude, but the end point never returned to the control state. 

## 4. Discussion

Autism is a neurodevelopmental disease, and this study was designed to examine hormonal signaling in cell growth rather than examining the mechanism of hormone action. Our principle finding is that B-lymphocytes derived from autistic individuals have a different response to E2, DHT, and DDE when compared to control subjects. We find that the biggest difference between the AUT and the internal/external control cells is in their response to DHT, where they show very little growth modulation and do not upregulate their mitochondrial levels (see Figures 1 and 2). AUT cells had much smaller changes in the amplitude of both growth depression and mitochondrial number upregulation, and higher levels of DHT were needed to change mitochondrial levels in AUT. 

We find this lack of response to DHT by the AUT B cells very interesting, as the same cells respond to E2 incubation by increasing their mitochondrial levels. Thus, their lack of response to DHT is not due to an inability to increase their mitochondria numbers but some other unknown factor suppressing this response. Such insensitivity to DHT could represent a desensitization of the normal signaling pathway(s) due to *in utero* exposure [45, 46]. The effects we observe in using DHT are unlikely to be direct androgenic effects, given the resemblance of the E2 and DHT “U”-shaped growth curves. It is more likely that DHT converts into an androgen with KDs of 6 and 2 nM for the ER*α* and ER*β* receptors, respectively [47].

The hormone disruptors we used (phthalate DEHP, HO-PCB, and nonylphenol bis-phenol) had similar effects as E2, DHT, and DDE but at lower concentrations. AUT cells were less sensitive to the effects of these hormone disruptors than are the external controls. However, the ability of AUT to increase their mitochondria numbers, when challenged with low levels of these hormone disruptors, was always lower than the controls. This again suggests an underlying mitochondrial deficit in the AUTs.

### 4.1. ASD, Male Brains, and Fetal Testosterone

The extreme male brain (EMB) theory of autism was initially formulated by Hans Asperger in 1944. He wrote “The autistic personality is an extreme variant of male intelligence. Even within the normal variation, we find typical sex differences in intelligence. In the autistic individual, the male pattern is exaggerated to the extreme” [48]. Baron-Cohen forwarded a new theory of the psychology of sex differences in the 1990s (the Empathizing-Systemizing theory) and related this to Asperger's EMB theory [45]. The Cambridge Longitudinal Foetal Testosterone Project examined *in utero* testosterone levels and child developmental parameters. It showed that fetal testosterone (fT) is negatively correlated with social and language development but is positively correlated with a number of autistic traits [46], suggesting a role for high androgen levels in ASD.

The 2D : 4D ratio in females appears to be a robust proxy of prenatal androgen exposure but is less robust in males [49]. Children with ASD and also children with ADHD/ODD have significantly lower 2D : 4D ratios than controls [50]. The correlation is very strong when comparing autistic girls to unaffected girls [51]. Handedness is also tied to the 2D : 4D ratio [52], and *in utero* androgen exposure has long been believed to predispose to left-handedness. Recent evidence shows that fT levels in amniotic fluid predicts both language lateralization and handedness [53]. This adds further support for a role of fT in ASD; as in addition to having a low 2D : 4D ratio, the degree of non-right-handedness is 65% in ASD [54, 55], compared to 10–12% of the general population.

### 4.2. Sexual Brain Sculpting

Estrogen and androgen steroids are involved in the process of brain sculpting that generates sexual dimorphic male/female brains. The largest known cognitive sex difference in human males and females is spatial ability [56], with 3D mental rotation tasks showing the greatest gender difference [57]. A recent study shows that ASDs significantly outperform matched controls in many complex 3D mental rotation tasks [58]. Testosterone has direct effects via the androgen receptor and indirect effects following its enzymatic conversion to dihydrotestosterone (DHT) and estradiol (E2), with both converting enzymes present in neural tissue. DHT is also a prohormone, and two enzymes generate the neurosteroids 5*α*-androstan-3*α*,17*β*-diol and 3*β*,17 *β*-diols [59]. The 3*β*-diol is an agonist for ER*β* [60], and thus some DHT actions in brain are mediated by conversion to 3*β*-diol and subsequent ER*β* activation [61].

Sex hormone signaling disorders inform us of the extent that estrogen, testosterone, and DHT have on converting the default “female” brain into that of the “male” brain. 46XY individuals with complete androgen insensitivity syndrome (CAIS) lack a functioning androgen receptor, and their tissues are unable to respond to testosterone/DHT. They are conventionally feminine across a range of psychological tests [62], and 2D : 4D digit ratios are feminized [63]. 46XY individuals with complete 5-alpha-reductase-2-deficiency (5R2D) have low or zero DHT and slightly elevated T levels. They present as female at birth; however, the brain is masculinized even in individuals raised as girls [64]. Complete aromatase deficiency (AD) is very rare [65], but 46XYAD individuals who lack estrogen throughout development have brain masculinization [66]. 21-hydroxylase deficiency (21HD) results in testosterone/DHT overexposure *in utero* and virilization of females. 46XX21HD individuals [62] have a mildly masculinized spatial ability compared with unaffected control females, a low 2D : 4D ratio [67, 68], and a pronounced left-handedness bias [69].

### 4.3. Extreme Male Brain, Genotype, and DHT

It has long been known that men with androgenetic alopecia (AGA) have high levels of 5-alpha-reductase (5AR), total androgens, unbound/free androgens, and a high DHT : T ratio [70]. A genome scan looking for DHT susceptibility loci in 95 families where at least two brothers had early-onset AGA has been conducted. The AR gene in the Xp11-q25 region, long associated with AGA, had an NPL score of 2.67, but a second locus, 3q26, scored 2.69 [71]. This locus has been linked to ASD in two distinct groups. A locus at 3q26 was found to be highly linked to these conditions in a Finnish study of 38 families with high rates ASD. In a very large “Utah” ASD family there are 7 ASD males, 11 unaffected males, and 10 unaffected females [72], and the 3q26.31–q27.3 region was again an ASD locus. Thus, at least one region on chromosome 3 is implicated in AGA, a DHT-sensitive condition, and ASD, a putative androgenic sensitive condition.

This study was designed to give insight into the interaction of environment and genes with respect to ASD. A common womb constitutes part of the environment of the 10 AUT, Bro, and Sis, but not the Con. The selection of cells from unaffected twins also constitutes a selection bias. If a gestational ASD trigger is present in the 10 families, then by examining only unaffected brothers, we may be observing a “survivor effect.” The statistically significant difference found only between AUT and Bro could possibly be explained by the following: AUT represent a population susceptible to a familial gestational trigger, Bro represent a risk resistant population immune to this “theoretical” insult, and the external controls represent an at risk population never exposed to the trigger as they were formed in another womb.

There is increasing evidence that individuals with autism are more sensitive to reactive oxygen species (ROS), and that changes in this sensitivity may be important in understanding the pathophysiology of autism. In this regard, we find it to be interesting and important that exposure to hormone disruptors influences mitochondrial function, as upregulation of mitochondria number and function are important responses to present damage from ROS. In individuals with inherent genetic differential sensitivities to hormone disruptors, early environmental exposure may play an important role in either the etiology of the disorder, or in the development of a number of cellular and metabolic deficits associated with it. Recent publications from our lab and others have implicated thimerosal in similar metabolic pathways examined in this paper [73, 74]. The possibility of thimerosal and/or other neurotoxins interacting with the hormones and hormone disruptors presented here to yield the ASD phenotype is an area which we are currently investigating.

Finally, it is quite clear that B-lymphocytes are not normally present in brain and that autism is a neurological disease. Thus, our B-cell data does not directly inform us if estrogens, androgens, and hormone disruptors have differential effects on the development and growth of the brain in autistic individuals. However, we did examine the effects of E2, DHT, and DDE on human astrocytes and cortical neurons at concentrations where there was a clear differential effect on autistic B cells. We found that there were similar effects on cell growth and mitochondrial upregulation in astrocytes and neurons compared to those found in B-lymphocytes. We therefore think it is reasonable to postulate that the disruption of hormonal signaling we found in B-lymphocytes from individuals with autism could also be present in neurons and glial cells of these individuals.

## 5. Conclusion

B-lymphocytes from people with ASD exhibit a differential response to E2, DHT, and hormone disruptors in regard to cell growth, and mitochondrial number increases when compared to non-ASD siblings and external controls. Specifically, ASD B-lymphocytes show significantly less growth depression and less increase in mitochondrial number when exposed to these effectors. Normal human astrocytes and human cortical neurons exhibit responses similar to control B-lymphocytes with respect to cellular growth and mitochondrial response when exposed to these agents. While astrocytes and neurons from individuals with ASD were not tested in this study, the data here suggests that a response similar to what was seen in B-lymphocytes may occur in brain tissue in autistic individuals.

## Figures and Tables

**Table 1:** Effects of charcoal stripping on hormone levels of calf fetal serum.

Hormone	Progesterone ng/mL (nM)	Testosterone pg/mL (nM)	Estradiol pg/mL (pM)	Estrone pg/mL (nM)
Fetal calf serum	<0.20	472 (1.6)	0.4 (1.5)	14,247 (53)
Stripped	<0.20	<200	ND	13

Levels of hormones in unstripped/stripped fetal calf serum as determined by an independent laboratory, Texas Veterinary Medical Diagnostic Laboratory, College Station, TX, USA. ND: nondetected.

**Table 2:** Comparison of cell growth cumulative sum plots.

Drug	Abs	Ord	*r* ^2^	Actual observed slope ± 90% CI	Theoretical critical slope ± 90% CI	*P* < 0.01
E2	*AUT *	*Bro *	*0.999 *	*1.48–1.408 *	*1.216–0.18 *	**∗∗**
	*AUT *	*Sis *	*0.996 *	*1.488–1.444 *	*1.22–0.183 *	**∗∗**
	*AUT *	*CON *	*0.998 *	*1.692–1.574 *	*1.221–0.184 *	**∗∗**
	**Bro**	**Sis**	**0.999**	**1.033–0.996**	**1.238–0.192**	**—**
	**Bro**	**Con**	**0.9997**	**1.147–1.115**	**1.239–0.193**	**—**
	**Sis**	**Con**	**0.9984**	**1.149–1.079**	**1.243–0.196**	**—**

DHT	*AUT *	*Bro *	*0.9925 *	*2.457–2.139 *	*1.28–0.201 *	**∗∗**
	*AUT *	*Sis *	*0.9948 *	*2.575–2.297 *	*1.279–0.2 *	**∗∗**
	*AUT *	*CON *	*0.991 *	*1.911–1.637 *	*1.455–0.327 *	**∗∗**
	**Bro**	**Sis**	**0.9997**	**1.075–1.043**	**1.15–0.13**	**—**
	**Bro**	**Con**	**0.9991**	**0.791–0.753**	**1.308–0.268**	**—**
	**Sis**	**Con**	**0.999**	**0.747–0.711**	**1.307–0.267**	**—**

DDE	*AUT *	*Bro *	*0.9984 *	*1.749–1.639 *	*1.277–0.207 *	**∗∗**
	*AUT *	*Sis *	*0.9986 *	*1.853–1.753 *	*1.264–0.196 *	**∗∗**
	*AUT *	*CON *	*0.9988 *	*1.86–1.762 *	*1.372–0.277 *	**∗∗**
	**Bro**	**Sis**	**0.9998**	**1.075–1.053**	**1.186–0.155**	**—**
	**Bro**	**Con**	**0.996**	**1.12–1.012**	**1.288–0.24**	**—**
	**Sis**	**Con**	**0.983**	**1.046–0.959**	**1.272–0.232**	**—**

Table 2 shows comparison of the changes in fractional growth of the four cell types and three effectors and is based on Figure 2 cumulative sum plots. All fitted plots produced a linear trend where the rho value of the fit was <0.01. Columns 1–3 of Table 2 show the effector examined and the pair of cell types whose correlation is compared. The *r* ^2^ statistic of the resultant slope is shown in the 4th column. The 5th column shows the 90% confidence interval of the generated slope. The 6th column indicates the theoretical 90% confidence interval around a slope of 1, calculated from the variance of each pair of cell type's response to the tested effector. The final (7th) column indicates if the growth response of the two cell populations in question differs at the *P* = 0.01 confidence level.

ASD: autism spectrum disorder; AUT: B cells from individual with ASD; Bro: B cells from brother of individual with ASD; CI: confidence interval; Con: control B cells from individual with no personal or family history of ASD; DDE: dichlorodiphenyldichloroethylene; DHT: dihydrotestosterone; E2: estradiol; LDH: lactate dehydrogenase; Sis: B cells from phenotypically normal sister of individual with ASD; XTT: 2,3-bis(2-methoxy-4-nitro-5-sulfophenyl)-2H-tetrazolium-5-carboxanilide; XTT/LDH: ratio of XTT/LDH indicative of mitochondrial function per cell.

**Table 3:** Comparison of changes in XTT/LDH ratio.

Drug	Abs	Ord	*r* ^2^	Measured slope ± 90% CI	Critical slope ± 90% CI	*P* < 0.01
E2	**AUT**	**Bro**	**0.952**	**1.579–1.1**	**1.443–0.327**	**—**
	**AUT**	**Sis**	**0.970**	**1.722–1.303**	**1.431–0.318**	**—**
	*AUT *	*CON *	*0.996 *	*2.245–2.032 *	*1.402–0.296 *	∗∗
	**Bro**	**Sis**	**0.995**	**1.181–1.05**	**1.569–0.359**	**—**
	**Bro**	**Con**	**0.971**	**1.748–1.327**	**1.537–0.339**	**—**
	**Sis**	**Con**	**0.987**	**1.514–1.257**	**1.516–0.333**	**—**

DHT	**AUT**	**Bro**	**0.848**	**1.291–0.641**	**1.504–0.358**	**—**
	**AUT**	**Sis**	**0.975**	**1.611–1.249**	**1.372–0.264**	**—**
	*AUT *	*CON *	*0.997 *	*2.144–1.959 *	*1.556–0.395 *	∗∗
	**Bro**	**Sis**	**0.941**	**1.604–1.074**	**1.519–0.311**	**—**
	**Bro**	**Con**	**0.874**	**2.382–1.28**	**1.723–0.434**	**—**
	**Sis**	**Con**	**0.985**	**1.547–1.269**	**1.502–0.379**	**—**

DDE	**AUT**	**Bro**	**0.942**	**1.522–1.021**	**1.432–0.311**	**—**
	**AUT**	**Sis**	**0.961**	**1.677–1.213**	**1.438–0.316**	**—**
	*AUT *	*CON *	*0.993 *	*2.215–1.936 *	*1.478–0.344 *	∗∗
	**Bro**	**Sis**	**0.996**	**1.182–1.064**	**1.504–0.337**	**—**
	**Bro**	**Con**	**0.973**	**1.775–1.361**	**1.545–0.364**	**—**
	**Sis**	**Con**	**0.985**	**1.538–1.267**	**1.556–0.367**	**—**

Table 3 shows comparison of the changes in XTT/LDH ratio of the four cell types and three effectors and is based on Figure 2 cumulative sum plots. All fitted plots produced a linear trend where the rho value of the fit was <0.01. Columns 1–3 of Table 2 show effector examined and the pair of cell types whose correlation is compared. The *r* ^2^ statistic of the resultant slope is shown in the 4th column. The 5th column shows the 90% confidence interval of the generated slope. The 6th column indicates the theoretical 90% confidence interval around a slope of 1, calculated from the variance of each pair of cell type's response to the tested effector. The final (7th) column indicates if the growth response of the two cell populations in question differs at the *P* = 0.01 confidence level. AUT and Con were the only 2 groups that showed a significantly different response to the 3 effectors (E2, DHT, and DDE).

ASD: autism spectrum disorder; AUT: B cells from individual with ASD; Bro: B cells from brother of individual with ASD; CI: confidence interval; Con: control B cells from individual with no personal or family history of ASD; DDE: dichlorodiphenyldichloroethylene; DHT: dihydrotestosterone; E2: estradiol; LDH: lactate dehydrogenase; Sis: B cells from phenotypically normal sister of individual with ASD; XTT: 2,3-bis(2-methoxy-4-nitro-5-sulfophenyl)-2H-tetrazolium-5-carboxanilide; XTT/LDH: ratio of XTT/LDH indicative of mitochondrial function per cell.

**Table 4:** Summary of differential hormone/hormone disruptor effects on B-cell growth.

Effector	Controls			AUT		
	Pseudo-*K*_*D*_1	Pseudo-*K*_*D*_2	Max XTT/LDH	Pseudo-*K*_*D*_1	Pseudo-*K*_*D*_2	Max XTT/LDH
DHT	4 nM	>20 nM	22.8%	10 nM**	>20 nM	13.0%**
E2	0.75 nM	>2.1 nM	23.9%	0.45 nM**	>2.1 nM	14.7%**
DDE	15 nM	220 nM	23.8%	40 nM**	>250 nM	15.6%

DEHP	*≈*100 pM	>100 *µ*M	18.4%	*≈*50 pM**	>100 *µ*M	14.5%*
Nonylphenol	10 pM	>100 *µ*M	29.4%	10 pM	>100 *µ*M	20.4%**
Bis-Phenol A	<100 pM	100 *µ*M	33.0%	<100 pM	>100 *µ*M*	29.1%*
HO-PCB	0.5 pM	>250 nM	22.0%	<0.5 pM**	>250 nM	15.6%**

Table 4 shows the concentration of hormone/hormone disruptor that defines 50% of the slopes of the *U*-shaped proliferation curves and maximum recorded XTT/LDH%. **P < 0.01 or *<0.05% for AUT versus controls (ANOVA and post hoc analysis [20]).

ASD: autism spectrum disorder; AUT: B cells from individual with ASD; DDE: dichlorodiphenyldichloroethylene; DEHP: di(2-ethylhexyl)phthalate; DHT: dihydrotestosterone; E2: estradiol; HO-PCB: hydroxylated polychlorinated biphenyls; *K*_*D*_: dissociation constant; LDH: lactate dehydrogenase; XTT: 2,3-bis(2-methoxy-4-nitro-5-sulfophenyl)-2H-tetrazolium-5-carboxanilide; XTT/LDH: ratio of XTT/LDH indicative of mitochondrial function per cell.
